# Factors Associated With Late Prematurity in the University Hospital of Valle Cali, Colombia During 2013–2014

**DOI:** 10.3389/fpubh.2020.00200

**Published:** 2020-07-10

**Authors:** Javier Torres-Muñoz, Carlos Alberto Jiménez-Fernandez, Rubi Rocio Ortega, Darly Janeth Marin Cuero, Diana Marcela Mendoza

**Affiliations:** ^1^INSIDE Research Group Department of Pediatrics Universidad del Valle, University Hospital of Valle, Cali, Colombia; ^2^Department of Pediatrics, University Hospital of Valle, Cali, Colombia; ^3^Faculty of Health, Medicine and Surgery Program, School of Medicine, Universidad del Valle, Cali, Colombia

**Keywords:** late premature, associated factors, premature, morbidity, newborn

## Abstract

**Introduction:** The birth rate of late premature babies has been increasing in recent years, composing now 75% of all premature births. This growing trend can be explained by different demographic transformations such as an increase in the demand for infertility treatments, older maternal age and the higher incidence of multiple pregnancies, cesarean sections, and labor induction. These premature babies contribute 30% to the global neonatal mortality rate.

**Objective:** To identify the factors associated with late prematurity at the Hospital Universitario del Valle during the years 2013–2014.

**Methodology:** Case and control design, 424 patients, 212 cases and 212 controls participated. Cases were defined as newborns with gestational age between 34 and 36 weeks and 6 days old. For the analysis, logistic regression models were developed and association forces (OR) were determined.

**Results:** A univariate analysis shows that the proportion of teenage pregnant women corresponds to 22.64%. Bivariate analysis shows the maternal morbidity due to hypertensive disorders was 1.6 times higher (95% CI 1.06–2.63), the obstetric alterations in 2.9 times (CI of 95% 1.56–5.44), late preterm infants require more oxygen support 3.26 times (95% CI 1.76–6.03). After adjusting the model, it was found that late premature infants have a 3-fold probability of requiring some resuscitation maneuver (ORa 3.23 95% CI 2.09–4.99), birth is higher by cesarean section by 4.17 times (ORa 4.17 IC 95% 2.50–6.98), maternal morbidity was higher in 1.37 times (ORa 1.37 95% CI 1.14–1.65). The morbidity of the newborn was greater, close to the statistical significance for late premature infants in 1.26 times (ORa 1.26 95% CI 0.97–1.64).

**Conclusions:** Late premature births in this study show a higher probability of developing morbidity, have a greater opportunity to be born by cesarean section, are products of mothers with morbidity (specifically hypertensive disorders), and require further resuscitation with a need of early obstetric intervention.

## Introduction

The premature delays correspond to about 74% of all premature births and 8% of total births (1). The American Academy of Pediatrics and the American College of Obstetricians and Gynecologists (ACOG) have defined late preterm infants as those born between the 34th and 36th weeks and 6 days of gestation, and have emphasized that these newborns are premature and as such, are at risk of medical complications related to immaturity. They are physiologically and metabolically immature and have a limited compensatory response capacity against extra-uterine changes compared to those born at term, which determines a high risk of morbidity and mortality (2).

As time progresses, care for the newborn evolves. However, late premature babies are today a cause of special interest, since they constitute a large percentage of neonatal mortality (2). Physiological, anatomical and metabolic deficiencies predispose these children to develop complications in the short and long term, with risk of resuscitation at birth twice as large compared to term infants (2).

Respiratory morbidity in late preterm infants has a high prevalence, with pathologies such as respiratory distress syndrome and transient tachypnea of the newborn, which lead to the need for resuscitation at birth, the supply of oxygen. and even ventilatory support (2). The current explanations for this pathologies are:

- Reduction of central chemosensitivity, which makes the body more tolerant of high concentrations of CO_2_.
- Immaturity of brain development, which leads to a poor response to physical and metabolic stress.
- Disruption of lung maturation, between the terminal and alveolar sac phase, which causes functional deficiencies of surfactant and inappropriate water management in the lung.

In these babies, the generated systemic failures decrease the functionality of the hypothalamus, hormonal concentrations and fat stores, contributing to instability in body temperature (hypothermia). Similarly, there are alterations in the suction-swallowing-breathing coordination, poor gastrointestinal motility, and low motor tone that produce failures in suction and swallowing processes, poor weight gain, dehydration and increased risk of death (3, 4).

Late preterm infants have an increased risk of developing neurodevelopmental difficulties by 7 years of age, as well as poor performance on standardized tests and developmental delay when compared to term children^.^(1). This is because approximately 50% of the increase in cortical volume occurs between weeks 34 and 40 of gestation, and it is considered that between weeks 36 and 40 there is an exponential increase in gray matter and myelination of white matter. This explains why they present 36% more risk of developmental delay or disability (5, 6).

There is a marked tendency in the literature to develop research focusing mainly on the comparison between late preterm infants and term infants, regarding the complications developed in each group. Various papers have been published on the associated risk factors, such as multiple pregnancies, advanced maternal age, maternal obesity, and premature rupture of membranes (5–7).

The impact of multiple pregnancies in late preterm infants has been widely evaluated. Most multiple pregnancies have the outcome of late premature birth, and are also associated with a series of fetal and maternal complications not related to prematurity, such as twin-twin transfusion syndrome, intrauterine growth restriction and the development of preeclampsia (6, 7).

The health risks for both the mother and the infant that are related to advanced maternal age are not well-documented. However, advanced maternal age contributes to an increase in cases of gestational arterial hypertension (6). Gestational arterial hypertension is a frequent cause of intrauterine growth restriction (RCIU), so premature delivery may be necessary (8). Also, this factor is associated with chromosomal abnormalities, pregnancy disorders, chronic diseases, the need for the use of assisted reproduction technology (ART), multiple births and cesarean sections, all of which may contribute to the global increase in premature births (7, 8).

Ultrasound fetal imaging to determine gestational age can be difficult in obese and overweight women. In these pregnancies, maternal obesity along with fetal macrosomia can lead to an overestimation of gestational age (7). This lack of precision can make it difficult for health professionals to make decisions, especially under adverse maternal conditions where there is a need to determine the optimal time of birth. Both fetal macrosomia and large neonate for gestational age are associated with an increased risk of traumatic birth and associated complications, which is why childbirth is brought forward in time through cesarean section or induction of birth. Maternal obesity is strongly associated with gestational diabetes, type 2 diabetes, gestational hypertension, cardiovascular disorders and many other chronic maternal diseases, all of which are risk factors for late premature births.

Premature rupture of membranes is responsible for one-third of premature births, and is another factor that contributes to the increase in late premature births (9). Choriodecidual infection is recognized as a fundamental pathophysiological mechanism of premature rupture of membranes and late premature delivery, as well as low socioeconomic status, smoking, sexually transmitted infections (STIs), multiple pregnancies, vaginal bleeding, and polyhydramnios (10). The practical guidelines published by ACOG recommend the use of tocolytics and corticosteroids for the treatment of premature labor until 34 weeks of gestation (11). When premature rupture of membranes occurs after 34 weeks, premature birth seems to be the most prudent strategy. The premature onset of labor, due to the RPM, represents a RR of three times greater and an adjusted OR of nine times greater, for the admission of neonates to intensive care units (12).

## Materials and Methods

### Ethical Considerations

The respective approval of the Ethics Committee of the Hospital Universitario de the Valle and the Universidad del Valle for the realization of this study was fulfilled.

Pregnant women who agreed to participate in the study gave authorization by an informed consent document, designed following the required ethical regulations.

### Design

An analytical observational case and control study conducted in Cali, Colombia, at the Hospital Universitario del Valle, which is a public institution providing level III services to mainly a low-income population, during the 2013–2014 period in Santiago de Cali. Newborns between 34 and 36 weeks of gestational age calculated by the Ballard test, selected at birth in the institution during the study period, as well as newborns that were defined as controls according to the selection criteria: born between week 37 and 40 of pregnancy in the institution. If they presented any morbidity, they entered intensive care. Exclusion criteria: Infants with major congenital malformations and mothers who would not accept to participate in the study.

To calculate the sample size, the Schlesselman formula was used for case-control studies, and taking into account the criteria of α = 0.05 and β = 0.10. For the estimation of the sample size an occurrence of late preterm of 15% was considered. The aim was to obtain an OR of 3 and a case-control ratio 1: 1 (taking into account a loss of participants of 20% due to incomplete information). Under these considerations, a sample size of 448 infants was obtained, where 224 correspond to the case group and 224 correspond to the controls. The cases were selected by intentional sampling; newborns that met the study inclusion criteria were selected. We searched the clinical records of the database that manages the Epidemiology area of the Hospital Universitario del Valle of all births from January 2013 to December 2014. The controls were selected from the birth and cesarean section registry of the Obstetrics-Gynecology Unit. Infants from 37 to 42 weeks of gestation were identified, and each case was paired with a control with the nearest gestational age. Infants with any morbidity entered the neonatal intensive care unit for the treatment according to the institution's protocols.

The data obtained from the clinical history, both the cases and the controls, were entered into an Epi Info version three data collection program, information that was subsequently exported to the STATA 13 software, for statistical analysis.

### Analysis Plan

The categorical variables generated were examined in an exploratory univariate analysis in which absolute frequencies were expressed, followed by a bivariate analysis applying the Chi-square test or Fisher's exact test, as appropriate. The strength of the association (OR) and its 95% confidence intervals (95% CI) were determined between the dependent and independent variables.

The respective adjusted OR of each group of variables was determined, once analyzed and ruling out possible interactions and confusion effects between the different factors and covariates considered. To adjust a multiple logistic regression model was implemented. To select the variables included in each of the models, the procedure *stepwise* of successive steps forward (*Forward)* was used.

## Results

The general characteristics of the population are described in Table 1.

**Table 1:** Clinical and sociodemographic characteristics of puerperal women with late preterm infants and term infants.

**Variable**		**Frequency**	**Percentage**
Age of the Mother (years)	<19	96	22.64
	19–35	286	67.45
	>35	42	9.91
Ethnicity of the mother	Mestizo	258	60.85
	Afro	149	35.14
	Indigenous	17	4.01
Level of schooling of the mother	None	38	9.00
	Primary complete	222	52.61
	Secondary complete	130	30.81
	Technical	32	7.50
Marital status of the mother	With partner	301	70.99
	Without partner	123	29.01
Current pregnancy desired	Desired	145	34.69
	Not desired	273	65.31
Number of prenatal controls performed by the mother	Prenatal controls <3	416	98.35
	Prenatal control 4–7	5	1.18
	Control >8	2	0.47
Gestacional age of the newborn	34	46	10.85
	35	65	15.33
	36	105	24.76
	Term	208	49.06
Birth way of the newborn	Vaginal	137	32.31
	Cesarean section	287	67.69
Maternal morbidity during pregnancy	None	114	26.89
	Hypertensive disorder	242	57.08
	Obstetric	68	16.04
Newborn weight at birth	<1,500	8	1.90
	1,500–2,500	173	41.00
	>2,500	241	57.11
Newborn weight type	Small	41	9.67
	Suitable	351	82.78
	Large	32	7.55
Type of newborn morbidity	None	254	59.91
	Neurological	52	12.26
	infectious	118	27.83
Primary maternal parity	Primip1	166	39.15
	Multiparous 2–5	248	58.49
	Large multiparous ≥6	10	2.36
Gender of the newborn	Female	213	50.35
	Male	210	49.65

Table 1 corresponds to the univariate analysis of maternal and newborn variables in cases and controls, were we evaluated the frequency of events analyzed. It was evidenced that 22.64% of the women were teenagers and 35.14% had Afro ethnicity. Overall, 65% did not want a pregnancy and 98% had inadequate prenatal control. Furthermore, hypertensive disorders represented the highest proportion of maternal morbidity and infections in the newborn.

### Bivariate Analysis

A bivariate analysis using the Chi square test or the Fisher exact test, as appropriate. The strength of the association (OR) and its 95% confidence intervals (95% CI), between the dependent variable and the independent ones, were determined. With this methodology, different associations were identified for each group of variables (Table 2).

**Table 2:** Comparative analysis between late preterm infants and term infants.

**Variable**	**Category**	**Cases (212)%**	**Controls (212)%**	***P***	**OR 95% CI**
Mother's age	<19 years	49(23.11)	47 (22.17)		1
	19–35	140(66.04)	146 (68.87)	0.72	0.91 (0.57–1.46)
	> 35	23(10.85)	19 (8.96)	0.68	1.16 (0.56–2.40)
Ethnicity of the mother	Mestizo	125(58.96)	133 (62.74)		1
	Afro	81 (38.21)	68 (32.08)	0.25	1.26 (0.84–1.89)
	Indigenous	6 (2.83)	11 (5.19)	0.29	0.58 (1.20–1.61)
Mother's school level	None	21 (9.91)	17 (8.10)		1
	Primary complete	113 (53.30)	109 (51.90)	0.61	0.83 (0.42–1.67)
	Secondary complete	62 (29.25)	68 (32.38)	0.41	0.73 (0.35–1.52)
	Technical	16 (7.55)	16 (7.62)	0.66	0.80 (0.31–2.07)
Marital status of the mother	With partner	146 (68.87)	155 (73.11)		1
	No partner	66 (31.13)	57 (26.89)	0.33	1.22 (0.8–1.87)
Current pregnancy desired	If wanted	65 (30.95)	80 (38.46)		1
	Did not want	145 (69.05)	128 (61.54)	0.10	1.39 (0.93–2.08)
Method of birth	Vaginal	48 (22.64)	89 (49.98)		1
	Cesarean section	164 (77.36)	123 (58.02)	0.00	2.47 (1.62–3.76)
Maternal morbidity during pregnancy	None	44 (20.75)	70 (33.02)		1
	Hypertensive disorders	124 (58.49)	118 (55.66)	0.02	1.67 (1.06–2.63)
	Obstetric events	44 (20.75)	24 (11.32)	0.00	2.91 (1.56–5.44)
Newborn weight type	Small	28 (13.21)	13 (6.13)		1
	Adequate	167 (78.77)	184 (86.79)	0.01	0.42 (0.21–0.84)
	Large	17 (8.02)	15 (7.08)	0.18	0.52 (0.20–1.36)
Newborn resuscitation maneuver	Positive pressure ventilation	130 (61.32)	190 (89.62)		1
	Oxygen at free flow	38 (17.92)	17 (8.02)	0.00	3.26 (1.76–6.03)
	Cardiac massage	42 (19.81)	5 (2.36)	0.00	12.27 (4.73–31.86)
	Orotracheal intubation	2 (0.94)	0 (0)	0.00	3.09 (1.93–4.98)
Newborn morbidity type	None	100 (47.17)	154 (72.64)	0.00	0.66 (0.51–0.84)
	Neurological	31 (14.62)	21 (9.91)		1
	Infectious	81 (38.21)	37 (17.45)	0.00	1.86 (1.47–2.34)
Primary maternal parity	Primipara 1	87 (41.04)	79 (37.26)		1
	Multiparous 2–5	116 (54.72)	132 (62.26)	0.26	0.79 (0.53-1.18)
	Great multiparous 6 or more	9 (4.25)	1 (0.47)	0.04	8.17 (1.0–65.96)

Table 2 corresponds to bivariate analysis of cases and control, identifying significant results associated with late prematurity. Late preterm newborns required a greater chance of resuscitation (Oxygen 3.26 times, cardiac massage 12.27 times and orotracheal intubation 3.09 times). The mothers showed a higher probability of 2.91 times of obstetric pathology (such as pelvic disproportion, multiple pregnancy and the threat of preterm birth), and births by cesarean section by 2.47 times. Moreover, there was a greater maternal morbidity due to hypertensive events by 1.67 times in mothers of late preterm newborns.

### Final Model

In the selection process of the predictor variables, the results of the bivariate analysis were taken into account, using the variables that indicated a significant association (0.02 alpha) with the event, to subsequently apply the logistic model with the selection technique of forward variables (*Forward*).

Table 3 shows the results posterior to the adjustment in a multiple logistic regression model. There is a statistically significant association of late prematurity with the presence of maternal morbidity during pregnancy (specifically when presenting with hypertensive disorders and obstetric alterations) (ORa 1.37 95%IC 1.14–1.65) and birth via cesarean section (ORa 4.17 95% CI 2.50–6.98). Also, late preterm infants have a greater chance of requiring resuscitation maneuvers (ORa 3.23 95% CI 2.09–4.99) and have a close to statistical significance a greater opportunity to present neonatal morbidity (ORa 1.26 95% CI 0.97–1.64).

**Table 3:** Results posterior to the adjustment in a multiple logistic regression model.

**Variable**	**Adjusted OR**	***P***	**(95% CI)**
Newborn resuscitation maneuver	3.23	0.00	2.09–4.99
Newborn Birth route	4.17	0.00	(2.50–6.98)
Maternal morbidity during pregnancy	1.37	0.00	(1.14–1.65)
Type of newborn morbidity born	1.26	0.07	(0.97–1.64)

## Discussion

Reports of premature births in the world are close to 12%, of which 71% correspond to late premature babies, 12.6% moderate premature babies, 10.1% early premature babies, and 5.9% extreme premature (13, 14). In Colombia, the prematurity rate represents a 20%, much higher than reported in other high-income countries (15). Dimitriou et al. in Greece (16) investigated morbidity determinants in 548 late preterm infants, through a prospective cohort study, finding that 165 of the infants had morbidity during hospitalization associated with childbirth. The most frequent morbid conditions were respiratory distress (25.4%) and hyperbilirubinemia that required phototherapy (25.2%). Early gestational age (OR 7.32 for 34 weeks and 3.05 for 35 weeks gestation) and Small PEG for gestational age (OR 2.69), were recognized as factors associated with a significant impact on neonatal morbidity. In Turkey, Özlem et al. (17) (2005–2007) developed a study that included 252 late preterm infants, presenting a mortality rate of 2.3%, whilst no term neonates died in the same period. When compared to term infants, late preterm infants were 11 times more likely to develop respiratory failure, 14 times more likely to have feeding problems, 11 times more likely to have hypoglycemia, three times more susceptible of being readmitted and 2.5 times more likely to require re-hospitalization.

Likewise, in a prospective multicenter control case study in Uruguay, Moraes et al. (18) described that respiratory distress syndrome had a statistically significant difference in late preterm infants with OR 4.57 (1.95–10.82) but not for those of 37 weeks. There were 19 cases of respiratory distress (26.3%) of which 17 (23.6%) corresponded to transient tachypnea, a case of hyaline membrane disease and a case of severe pulmonary hypertension (1.38%). Also, more metabolic disorders, hypoglycemia, hypothermia and jaundice were observed with statistical significance. Moreover, in Chile, Schonhaut et al. (19), studied neonatal morbidity in late preterm infants, which corresponded to 5.12% of live births of the year 2009. Premature infants have an increased risk of presenting respiratory distress syndrome (RR 17.3), apnea of prematurity (15.7) and third hypothermia (RR 10.8). In the present study, the probability of late prematurity was associated 3.23 times to higher requirement of resuscitation and 1.26 times (near the statistical significance) of developing early neonatal morbidity. Thus, as mentioned by previous publications and by this current investigation, late premature babies show a higher probability of requiring reanimation, with an ORa 3.23 (95% CI 2.09–4.99) and have a greater opportunity (close to statistical significance) of presenting neonatal morbidity (ORa 1.26 95% CI 0.97–1.64).

Furthermore, various investigations associate (20, 21) the development of morbidities during pregnancy with the probability of having a preterm birth. These associated morbidities were gestational diabetes and maternal hypertensive disease. The present study analyzed the factors significantly associated with late prematurity, such as morbidities of the mother (hypertensive disorders) and obstetric complications (disproportion pelvic head, multiple pregnancies, and the threat of preterm birth), that were correlated with an increased opportunity of 1.37 times of premature birth (95% CI 1.14–1.65). It is important to note that these factors can be recognized and identified early in the pregnancy, which indicates a way to prevent late prematurity.

A study with an ecological design (22) carried out in developed countries showed that places of high rates of late premature and premature births are associated with low rates of stillbirths and neonatal mortality. The authors consider that when cesarean birth is required due to medical indications in the preterm, they are generally beneficial since they had a significant risk of mortality. In this study, late premature births were also significantly associated by 4.17 times (95% CI 2.50–6.98) to birth by cesarean section, which was indicated for medical reasons, as many patients are referred to this institution of greater complexity due to their serious condition.

Some studies (23, 24) significantly associate late prematurity and increased neonatal morbidity to social factors such as low income or low schooling. In the present study, no association was observed to income or schooling, since the population that comes to this hospital as a whole belongs to low-income groups. Concerning their schooling, only 52% reached primary school, 31% achieved up to secondary school and 9% have no studies (in both groups). Furthermore, inadequate prenatal control (<3 prenatal controls) has also been described (21) as a factor associated with late prematurity. In the present study no association was evidenced, as 98% of the population treated in this institution in both groups did not receive adequate prenatal control.

Late premature babies were historically perceived with similar morbidity as full-term babies. However, several studies have shown that late premature babies manifest an increase in early complications much more frequently than full-term children. In this investigation, newborn morbidity was 1.26 times greater when compared to full-term children (ORa 1.26 95% CI 0.97–1.64), this is not significant due to sample size. However, it is pertinent to mention it, as other articles have investigated its importance in late prematurity. In Jobe (25) “A new disease: late preterm infants” and Davidoff MJ's publications (1), there is a growing concern for this group of infants, which is driven by the experience of health personnel directly involved, an increasing number of cases from 1990 to 2005, poor results related to health interventions in the first hospitalizations and an increase in medical care during its first year.

On account of the studies mentioned and the results observed in this research, the population of late premature infants has a greater association of short and long term complications compared to those born at term. Late premature infants have significant morbidity (its outcome non-lethal in the majority of investigations), are associated with disabilities, decreased quality of life and increased costs to the health services (25, 26).

A limitation of the study was using the medical records as a source, as some of these were incomplete, which did not allow for sufficient information in many of the variables analyzed. This significantly effects the result. Another limitation is that more than 90% of the population studied had low income, which did not allow social comparisons, as other studies have done.

## Conclusions

Birth of late premature infants has an important financial impact, derived from antepartum management, childbirth, neonatal treatment, and requirements of special medical care during their follow-up for several months. This forces an implementation of public health actions in the prevention of late preterm birth, such as improving access to adequate prenatal control. In this investigation, a very low percentage of prenatal control is observed and this consequently favors maternal morbidity. When maternal morbidity is not detected, it increases the chance of late preterm births with complications that require hospitalization, raises the probability of long-term effects and elevates financial costs for the health system.

## Data Availability Statement

The datasets generated for this study are available on request to the corresponding author.

## Ethics Statement

The studies involving human participants were reviewed and approved by Comité de ética de la Universidad del Valle; Comité de ética del Hospital Universitario del Valle. Written informed consent to participate in this study was provided by the participants' legal guardian/next of kin.

## Author Contributions

JT-M: study design and analysis and writing of the manuscript. CJ-F: study design and writing of the manuscript. RO: data collection and analysis. DC and DM: data collection. All authors contributed to the article and approved the submitted version.

## Conflict of Interest

The authors declare that the research was conducted in the absence of any commercial or financial relationships that could be construed as a potential conflict of interest.
